# Association Between Screen Time, Dietary Patterns, and Oral Health Among Children: A Cross-Sectional Study

**DOI:** 10.7759/cureus.81348

**Published:** 2025-03-28

**Authors:** Austin Robin, Vaishnavi Padmanabhan, Kavitha Swaminathan, Vignesh KC, Vivek K, Selvakumar Haridoss

**Affiliations:** 1 Dentistry, Sri Ramachandra Institute of Higher Education and Research, Chennai, IND; 2 Pediatric and Preventive Dentistry, Sri Ramachandra Institute of Higher Education and Research, Chennai, IND; 3 Pediatric and Preventive Dentistry, Sri Ramachandra Institute of Higher Education and Research, chennai, IND

**Keywords:** dental caries, dietary habits in children, pediatric oral health, screen time and children, sedentary behavior and oral health

## Abstract

Introduction: Screen time refers to the duration spent on digital devices, including televisions, computers, and mobile devices. Increased screen exposure among children raises concerns about its psychological, physical, and social health effects. Excessive screen time has been linked to poor dietary habits, reduced physical activity, and oral health issues such as dental caries. However, the association between screen time, dietary patterns, and oral health remains inadequately explored.

Aim: This study aimed to assess the association between screen time and children’s dietary patterns, oral hygiene practices, and oral health status.

Materials and methods: A cross-sectional observational study was conducted in a hospital-based setting in the Departments of Pediatric & Preventive Dentistry and Pediatrics over two months (December 2023-January 2024). A total of 353 children aged 6-13 years were recruited using convenience sampling. Screen time data were collected through parental interviews, while oral health status was assessed using the International Caries Detection and Assessment System II (ICDAS II) index. Dietary patterns were evaluated using a seven-day dietary recall. Data were analyzed using SPSS v23.0 (IBM Corp., Armonk, NY), with statistical significance set at p ≤ 0.05.

Results: Among the 353 participants, 187 (53.0%) were female. Nearly 66 (18.7%) children exceeded two hours of screen time per day on weekdays, while 276 (78.2%) exceeded this threshold on weekends. Only 60 (17.0%) reported brushing twice daily. The mean number of non-cavitated carious lesions was 0.7, while cavitated carious lesions averaged 3.8. Children who frequently watched screens while eating consumed approximately 22% of their meals in front of a screen. A statistically significant correlation was found between cavitated carious lesions and screen-related eating habits (p < 0.05), though the correlation coefficients were low.

Conclusion: The study identified a significant increase in screen time during weekends compared to weekdays. Higher screen use was associated with a greater number of cavitated carious lesions and total caries. Additionally, frequent screen use during meals was linked to unhealthy dietary patterns. Our findings highlight the importance of parental monitoring and structured screen time limits to encourage healthier dietary habits and improved oral hygiene among children.

## Introduction

Screen time refers to the duration spent passively viewing screen-based entertainment, such as televisions, computers, or mobile devices. It excludes interactive screen-based activities that require physical movement or engagement. The rapid advancement of digital media has led to an increase in screen exposure among children, affecting not only adolescents but even infants. The increasing screen time among children has emerged as a public health concern due to its association with psychological, physical, and social health issues [1,2].

Parental lifestyles play a significant role in shaping children's screen time habits. Extended screen exposure has been linked to negative outcomes, including poor dietary habits, reduced physical activity, obesity, and sleep disturbances [3]. According to the World Health Organization (WHO), infants under one year should not be exposed to screens, while children aged two to five years should have their screen time restricted to no more than one hour per day [4]. Similarly, the Indian Academy of Pediatrics recommends that children under two years old should avoid screen exposure entirely, with controlled usage for older children. Despite these guidelines, the prevalence of excessive screen time continues to rise, often compounded by sedentary behaviors, unhealthy dietary habits, and parental work schedules that limit supervision [5,6].

Excessive screen time is associated with an increased consumption of sugary foods and frequent snacking, both of which contribute to dental caries and periodontal disease. Studies have shown that children who spend extended periods on digital devices are more likely to engage in distracted eating, consume high-calorie, low-nutrient foods, and experience reduced parental supervision of oral hygiene routines [7,8]. The advertising industry also plays a crucial role, as exposure to food advertisements significantly influences children's food choices, increasing the preference for sugar-rich, carbohydrate-heavy diets [9,10].

Oral health issues linked to dietary patterns include dental caries, enamel erosion, gingival inflammation, and malocclusion. Poor oral hygiene practices, combined with increased screen exposure, further exacerbate these problems [11,12]. The impact of these factors extends beyond oral health, influencing overall well-being and psychological behavior in children [13].

Although several studies have explored the impact of screen time on children's physical health, sleep patterns, and obesity, limited research has focused on its direct relationship with oral health behaviors and dietary habits. This study is novel as it comprehensively examines the correlation between screen time duration, dietary patterns, and oral health status in children aged 6-13 years, using the International Caries Detection and Assessment System II (ICDAS II) index for caries assessment. Unlike previous studies that primarily focus on general health risks, our study uniquely assesses the behavioral aspects of screen time-related eating habits and their impact on caries prevalence. The findings will contribute to developing targeted interventions aimed at reducing screen-related oral health risks.

## Materials and methods

Study design and setting

This observational, cross-sectional study was conducted in a hospital-based setting in the Department of Pediatric & Preventive Dentistry and the Department of Pediatrics, Sri Ramachandra Institute of Higher Education and Research (SRIHER), Chennai, India. The study was carried out for two months, from December 2023 to January 2024.

Ethical considerations

The study was approved by the Institutional Ethical Committee (IEC) under ethical approval number CSP/23/SEP/136/837. Written informed consent was obtained from the parents or legal guardians of all participants, and written assent was obtained from children above seven years of age before data collection.

Objectives

Primary Objective

The primary objective of this study is to evaluate the association between screen time duration and oral health status, particularly in terms of cavitated and non-cavitated carious lesions using the ICDAS II index.

Secondary Objectives

The secondary objectives of this study are to analyze the correlation between screen time and dietary habits, particularly screen-based eating behavior, and its association with total meal frequency and cariogenic food consumption; assess the relationship between screen time and oral hygiene practices, including brushing frequency, nighttime brushing, and parental supervision of oral care; and investigate the influence of socioeconomic status (SES) on screen-time behaviors, dietary habits, and caries prevalence.

Sample population

Participants were recruited using a convenience sampling method due to practical feasibility and accessibility within the hospital-based setting. Given the nature of the study, random sampling was not feasible, as participation required parental consent, face-to-face interviews, and a seven-day dietary recall, which introduced logistical challenges. Additionally, as the study involved direct oral examinations and diet history documentation, recruitment was limited to children attending routine dental and pediatric check-ups. Convenience sampling allowed for the efficient collection of data within a short study duration (two months) while ensuring an adequate sample size for statistical analysis. The sample size was estimated using the formula n = 4pq/L², based on the prevalence of screen time exceeding two hours per day among children, as reported in a previous study by Yilmaz et al. [14]. According to their findings, approximately 54% of children exceeded this screen time threshold. Based on this prevalence, the minimum required sample size was calculated as 341 participants, but a total of 353 children were included in the study.

Inclusion criteria

Children aged 6 to 13 years who report for routine check-ups are eligible for inclusion in the study.

Exclusion criteria

Children with physical or developmental disabilities, neurological or psychological disorders, systemic illnesses, and those whose parents did not provide consent for the study were excluded from the study.

Data collection and examiner calibration

Demographic details were recorded, and screen time data were collected via parental interviews. The questionnaire assessed the type of devices used, average screen time on weekdays/weekends, and screen use during meals. An intraoral examination was performed to assess oral health, with caries status recorded using the ICDAS II index. Dietary habits were evaluated using a seven-day dietary recall.

The data collection was conducted by a single trained examiner (principal investigator). Examiner calibration was performed using 10 randomly selected patients, where the ICDAS II scoring was repeated after two weeks to assess intra-examiner reliability (Cohen’s Kappa = 0.89). Socioeconomic status data were obtained via parental interviews using a standardized questionnaire, while oral hygiene practices were collected through self-reported data from parents and children.

This study did not differentiate between passive screen use (e.g., watching TV) and interactive screen use (e.g., gaming and online learning). The total screen time was recorded as cumulative hours per day across all devices. Future studies should consider evaluating the differential impact of passive and interactive screen exposure on oral health outcomes.

Data on oral hygiene practices were collected through parental interviews and self-reported questionnaires administered to both parents and children. The questionnaire assessed factors such as brushing frequency (once or twice daily), use of additional oral hygiene aids (floss and mouthwash), nighttime brushing habits, and supervision of brushing by parents. Participants were categorized into those who brushed once daily and those who brushed twice daily, with additional data on whether they skipped nighttime brushing.

Socioeconomic status (SES) was assessed using a structured questionnaire based on the modified Kuppuswamy Socioeconomic Scale, which considers parental education, occupation, and family income. Participants were classified into five SES categories: Level 1 (Upper), Level 2 (Upper middle), Level 3 (Lower middle), Level 4 (Upper lower), and Level 5 (Lower). This classification allowed for the analysis of how socioeconomic factors influence oral hygiene practices and caries prevalence.

Data analysis

Statistical analysis was performed using IBM SPSS Statistics (version 23.0, IBM Corp., Armonk, NY). Descriptive statistics were reported as mean (SD) for continuous variables and n (%) for categorical variables. The unpaired t-test and ANOVA were used for comparisons between groups, while Chi-square tests analyzed categorical variables. A p-value ≤ 0.05 was considered statistically significant. The normality of data was assessed using the Kolmogorov-Smirnov test. As caries scores did not follow a normal distribution, non-parametric tests were applied. The Mann-Whitney U test was used to compare cavitated carious lesions between different screen time groups, and the Spearman correlation test was used to analyze the relationship between screen time and carious lesions.

## Results

**Figure 1:**
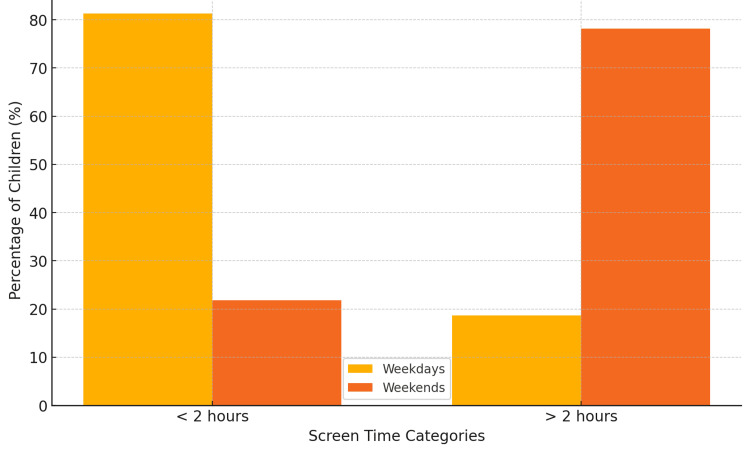
Screen time distribution on weekdays versus weekends This bar chart illustrates the percentage of children with screen time less than or greater than two hours on weekdays and weekends. The data shows a significant increase in screen time on non-working days.

Among the 353 participants, the mean age of the participants was 9.4 ± 2.1 years, and 187 (53.0%) were female. On weekdays, the average screen time among participants, n = 66 (18.7%), was 2.6 ± 1.1 hours per day, whereas on weekends, n = 276 (78.2%), it increased to 4.5 ± 1.3 hours per day (Figure 1). The average screen time among participants was 3.4 ± 1.2 hours per day, with a significantly higher duration on weekends compared to weekdays.

The Mann-Whitney U test was applied to compare cavitated carious lesions between screen time categories (Table 1).

**Table 1:** Comparison of cavitated carious lesions between screen time categories *Statistical significance at p ≤ 0.05.

Screen time category	Number of non-cavitated carious lesions (Mean ± SD)	p-value
Screen time on school working days		
<2 hours	3.8 ± 3.1	0.675
>2 hours	4.1 ± 3.6	-
Screen time on holidays		
<2 hours	2.7 ± 2.7	0.001*
>2 hours	4.1 ± 3.3	-

A comparison of cavitated carious lesions across different screen time categories showed a statistically significant difference on weekends (p = 0.001) but not on weekdays (p = 0.675).

Socioeconomic status and dental caries

Socioeconomic status was assessed using a standardized questionnaire. The majority of participants belonged to Level 2 (n = 217, 61.5%) and Level 3 (n = 88, 24.9%) socioeconomic status categories. Oral hygiene practices were self-reported by parents, with 60 (17.0%) children brushing twice daily and 343 (97.2%) using only a toothbrush and toothpaste. The mean number of cavitated carious lesions was higher among children from lower SES backgrounds compared to those from higher SES groups, though the difference was not statistically significant (p = 0.072). However, the correlation between SES and non-cavitated carious lesions was weak and non-significant (p > 0.05).

Association between screen time and caries prevalence

**Figure 2:**
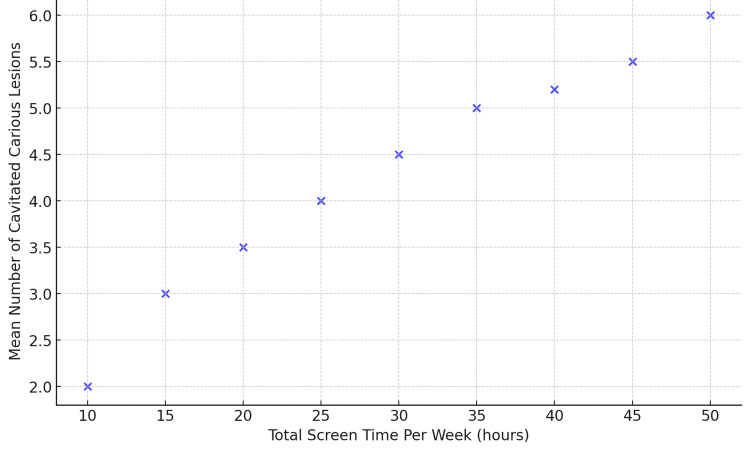
Correlation between total screen time and cavitated carious lesions This scatter plot displays the relationship between total screen time per week (in hours) and the mean number of cavitated carious lesions. The trend suggests a positive correlation between increased screen time and cavitated caries prevalence.

The mean number of non-cavitated carious lesions was 0.7, while the mean number of cavitated carious lesions was 3.8. The total caries burden (cavitated + non-cavitated lesions) had a mean of 4.53. A comparison of cavitated carious lesions across different screen time categories showed a statistically significant difference on weekends (p = 0.001) but not on weekdays (p = 0.675). A Spearman correlation analysis revealed a statistically significant correlation between total screen time and cavitated carious lesions (r = 0.140, p = 0.004) (Figure 2).

However, no significant correlation was observed for non-cavitated carious lesions (r = -0.072, p = 0.088), as shown in Table 2.

**Table 2:** Correlation of total screen time with cavitated and non-cavitated carious lesions Spearman's correlation test. *Statistically significant at p ≤ 0.05.

Variables		Total screen time (per week in hours)
Number of non-cavitated carious teeth	r value	-0.072
	p-value	0.088
Number of cavitated teeth	r value	0.140
	p-value	0.004*

Screen time, eating habits, and caries risk

Children who frequently used screens while eating consumed an average of 8.63 meals per week in front of screens, with 22% of their total meals occurring during screen use (Table 3).

**Table 3:** Correlation of cavitated and non-cavitated carious lesions with meals had while watching screen devices Spearman's correlation test. *Statistical significance at p ≤ 0.05.

Variables		Number of meals had while watching screen devices in a week	Percentage of meals had while watching screen devices in a week
Non-cavitated	r value	-0.040	-0.055
	p-value	0.230	0.151
Cavitated	r value	0.167	0.181
	p-value	0.001*	0.001*
Total caries	r value	0.142	0.148
	p-value	0.004*	0.003*

**Figure 3:**
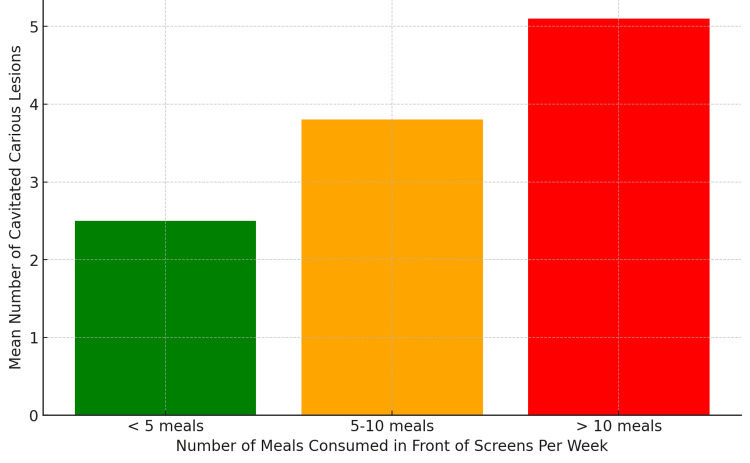
Eating habits and caries risk: meals consumed in front of screens This bar chart compares the mean number of cavitated carious lesions among children categorized based on the number of meals consumed while watching screens per week. The data indicate a significant association between frequent screen time eating and higher caries prevalence.

A statistically significant correlation was found between cavitated carious lesions and the number of meals consumed while watching screens (r = 0.167, p = 0.001). The prevalence of cavitated carious lesions in the study population was 71.1% (n = 251/353) based on ICDAS II scoring (Figure 3). A similar correlation was found between cavitated carious lesions and the percentage of meals consumed in front of screens (r = 0.181, p = 0.001). However, non-cavitated carious lesions did not show any statistically significant correlation with meal frequency during screen use (p > 0.05).

Although the correlation between screen time and cavitated carious lesions was statistically significant (p < 0.05), the correlation coefficient (r = 0.167) indicates a weak correlation. Similarly, the correlation between total screen time and total caries burden (r = 0.142, p = 0.004) also suggests a low-strength association, emphasizing that while screen time may contribute to increased caries risk, other factors such as dietary habits, oral hygiene practices, and fluoride exposure may also play critical roles in caries development.

Screen time and oral hygiene

The relationship between screen time and oral hygiene practices was analyzed. Among children with screen time < 2 hours/day, n = 48 (22.6%) reported brushing twice daily, while among children with screen time > 2 hours/day, only 12 (6.9%) brushed twice daily. This difference was statistically significant (p = 0.003), indicating that higher screen time was associated with poorer oral hygiene habits. Additionally, among high screen-time users, n = 89 (51.4%) reported occasionally skipping nighttime brushing, compared to n = 43 (20.2%) among low screen-time users (p = 0.012).

## Discussion

Children's screen time has significantly increased post-COVID-19, raising concerns about its impact on various health-related aspects, including sleep quality, physical activity, obesity, and eye strain. Several studies have investigated these effects, but limited research has focused on their relationship with oral hygiene and dietary patterns. The present study assessed the average screen time spent by children and its effects on oral health and dietary habits.

Screen time patterns: weekdays versus weekends

Previous studies have shown varying estimates of children's screen time. Tambalis et al. reported that 65.4% of children had screen time <2 hours/day, while 34.6% had screen time ≥2 hours/day [6]. Similarly, Beck et al. found that >60% of children spent up to 2 hours/day on television or computers [15]. In contrast, Garg et al. reported that 88.7% of children exceeded 2 hours of daily screen time [7]. Consistent with prior findings, our study demonstrated that 18.7% of children had screen time >2 hours/day on weekdays, while this percentage increased to 78.2% on weekends/holidays. This significant increase in screen time on non-working days suggests that leisure-time screen exposure contributes substantially to children's total screen duration.

Screen time and oral health

Several studies have highlighted the association between prolonged screen use and poor oral health outcomes. Yilmaz and Avci reported a strong correlation between longer screen time and high DMFT scores [14]. Similarly, Garg et al. found that screen usage ≥2 hours/day was significantly linked to higher DMFT/DEFT scores [7]. Asaka et al. also demonstrated that media time exceeding 1 hour/day was associated with an increase in treated teeth [16]. In line with these findings, our study revealed that cavitated carious lesions were more prevalent among children with screen time >2 hours/day compared to those with ≤2 hours/day. A statistically significant difference in carious lesions was observed on weekends (p = 0.001) but not on weekdays (p = 0.675). The lack of significance on weekdays may be attributed to structured school routines that limit snacking and screen exposure.

A possible explanation for the association between increased screen time and dental caries includes higher consumption of cariogenic foods while watching television, increased exposure to junk food advertisements, influencing children's dietary choices, and food pouching habits observed in children engrossed in digital media.

Although several studies have linked screen time to carious teeth, its specific impact on non-cavitated lesions remains unclear. Our study found no significant differences in non-cavitated lesions between screen time categories, reinforcing the need for further investigation.

Socioeconomic status and dental caries

Socioeconomic status (SES) plays a crucial role in dietary habits and oral health outcomes. Amudha et al. reported that children from higher SES backgrounds had a greater prevalence of dental caries, likely due to higher sugar consumption and frequent snacking [17]. Kapil et al. found that 91.1% of deciduous teeth in upper-middle-class children had dental caries, with 100% prevalence in lower-middle and upper-lower classes [18]. Our study aligns with these findings, demonstrating that cavitated carious lesions were more frequent in children with screen time >2 hours/day, particularly on holidays, across all SES levels. However, the association between non-cavitated carious lesions and SES was not statistically significant.

Screen time, dietary patterns, and oral hygiene

Screen exposure has been linked to dental neglect and poor oral hygiene practices [14]. Tsuchiya et al. found that excessive gaming was associated with inadequate toothbrushing behaviors [19]. Our study supports this observation, showing that only 17% of children brushed twice daily. This could be due to late-night screen use, leading to skipping bedtime brushing and falling asleep with devices, and neglecting oral hygiene.

Furthermore, children who watched >90 minutes of television daily had higher consumption of cariogenic foods (Silva et al.) [20]. Our study found a significant correlation between screen use during meals and cavitated carious lesions. These results align with Ghimire and Rao, who observed that children exposed to food advertisements consumed more sugary beverages and had higher caries prevalence [21].

Strength of association: screen time, diet, and caries

Our study found a significant correlation between both the cavitated carious lesions and the number of meals consumed while watching screens (r = 0.167, p = 0.001) and the percentage of meals consumed in front of screens (r = 0.181, p = 0.001). However, similar to Punitha et al., who reported only a slight increase in DMFT scores among children who snacked frequently, our study found no significant association between non-cavitated lesions and meal consumption patterns [22]. Doichinova et al. reported that excessive sugar consumption raised caries risk, while Zahara et al. found no correlation between sugary food intake and caries prevalence [23,24]. These discrepancies may be due to variations in food types and textures, differences in oral hygiene habits, and timing of sugar intake (with meals vs. between meals).

Our study found a high prevalence of dental caries, with 71.1% of children having cavitated carious lesions and 84.1% exhibiting some form of caries (cavitated or non-cavitated lesions). These findings align with previous reports indicating a high burden of dental caries among school-aged children, particularly in populations with increased screen exposure and poor dietary habits [24]. Future research should investigate preventive strategies such as structured parental interventions, dietary modifications, and screen-time regulations to mitigate caries risk in this age group.

Potential confounders such as parental education, fluoride exposure, and the availability of caretakers at home could influence children's oral health outcomes. While these factors were not controlled in this study, previous research suggests that higher parental education is associated with better oral hygiene practices, whereas limited supervision may contribute to unhealthy screen-time behaviors and dietary habits. Future studies should incorporate these variables to better understand their role in screen time and caries risk.

Limitations and future research

While our study provides valuable insights, certain limitations must be acknowledged. As a cross-sectional study, causality cannot be established, limiting the ability to assess the direct cause-and-effect relationships. Another limitation is the lack of salivary factor assessment, as the study did not evaluate salivary pH or buffering capacity, both of which play a crucial role in caries development. Furthermore, dietary history was self-reported by parents, which introduces the possibility of under-reporting sugary food intake, particularly for meals consumed at school or outside the home. Parental screen time was not assessed in this study, though it may influence children's screen habits. Future research should evaluate the relationship between parental and child screen behaviors and their impact on oral health. Future research should consider longitudinal studies with larger sample sizes, incorporate salivary biomarker assessments, and explore the combined effects of screen time, dietary habits, and sleep patterns on oral health outcomes to provide a more comprehensive understanding of these associations. Investigating the role of salivary biomarkers such as pH levels and buffering capacity could provide deeper insights into the mechanisms linking screen time and dental caries. Additionally, studies assessing the impact of screen time on sleep quality and its indirect effects on oral hygiene practices and diet patterns would help build a more comprehensive understanding of these associations. Further research should also examine preventive strategies, such as parental education programs aimed at reducing screen time during meals and encouraging structured screen time regulations to promote better oral health and nutrition among children.

## Conclusions

The present study identified a significant difference in screen time usage between weekdays and holidays, with 78.2% of children exceeding two hours of screen exposure on non-working days. A higher prevalence of cavitated carious lesions and total caries was observed among children with increased screen use. Additionally, frequent screen use during meals was associated with unhealthy dietary patterns, with 22% of all meals being consumed in front of screens. The findings underscore the necessity of parental monitoring and structured screen time regulations to mitigate the risks associated with excessive screen exposure, promoting healthier dietary patterns and optimal oral hygiene in children.

## References

[1] (2024). Guidelines on physical activity, sedentary behaviour and sleep for children under 5 years of age. https://www.ncbi.nlm.nih.gov/books/NBK541170/.

[2] Canadian Paediatric Society, Digital Health Task Force, Ottawa Ottawa, Ontario Ontario (2018). Erratum: screen time and young children: promoting health and development in a digital world. Paediatr Child Health.

[3] Lissak G (2018). Adverse physiological and psychological effects of screen time on children and adolescents: literature review and case study. Environ Res.

[4] Gupta P, Shah D, Bedi N (2022). Indian Academy of Pediatrics Guidelines on screen time and digital wellness in infants, children and adolescents. Indian Pediatr.

[5] Mark AE, Boyce WF, Janssen I (2006). Television viewing, computer use and total screen time in Canadian youth. Paediatr Child Health.

[6] Tambalis KD, Panagiotakos DB, Psarra G, Sidossis LS (2020). Screen time and its effect on dietary habits and lifestyle among schoolchildren. Cent Eur J Public Health.

[7] Garg N, Khatri A, Kalra N, Tyagi R (2023). The association of screen time with intake of potentially cariogenic food and oral health of school children aged 8-14 years-a cross-sectional study. J Clin Pediatr Dent.

[8] Shqair AQ, Pauli LA, Costa VP, Cenci M, Goettems ML (2019). Screen time, dietary patterns and intake of potentially cariogenic food in children: a systematic review. J Dent.

[9] Kelly B, Halford JC, Boyland EJ (2010). Television food advertising to children: a global perspective. Am J Public Health.

[10] AlSaffan AD, AlDayel E, AlZamami M, Satout A, AlMotairi Y, Pani SC (2017). A content analysis of food and beverage advertisements associated with Arabic children's videos on the internet and its relation to dental health. Saudi J Oral Sci.

[11] Morgan M, Fairchild R, Phillips A, Stewart K, Hunter L (2009). A content analysis of children's television advertising: focus on food and oral health. Public Health Nutr.

[12] Li Y, Lv MR, Wei YJ, Sun L, Zhang JX, Zhang HG, Li B (2017). Dietary patterns and depression risk: a meta-analysis. Psychiatry Res.

[13] Vadivel AS, Ann Tryphena ET, Gowri S (2024). Influence of diet and nutrition on oral health - a review. J Academy Dent Educ.

[14] Yilmaz N, Avci G (2022). Exposure to screen time and dental neglect. J Paediatr Child Health.

[15] Beck H, Tesler R, Barak S, Moran DS, Marques A, Harel Fisch Y (2021). Can health-promoting schools contribute to better health behaviors? Physical activity, sedentary behavior, and dietary habits among Israeli adolescents. Int J Environ Res Public Health.

[16] Asaka Y, Sekine M, Yamada M, Tatsuse T, Sano M (2020). Association of short sleep duration and long media use with caries in school children. Pediatr Int.

[17] Amudha S, Moses J, Vijayakumar M, Shankar P (2021). Prevalence of dental caries among different socioeconomic status and their treatment needs among 5-15-year-old school-going children in Maduravoyal area, Chennai. Int J Clin Pediatr Dent.

[18] Kapil D, Saraf BG, Sheoran N, Srivastava P, Singh S, Singh R (2023). To assess the prevalence of dental caries and its association with body mass index, socioeconomic status, dietary habits, and oral hygiene among 6-12-year-old children in Faridabad. Int J Clin Pediatr Dent.

[19] Tsuchiya M, Momma H, Sekiguchi T (2017). Excessive game playing is associated with poor toothbrushing behavior among athletic children: a cross-sectional study in Miyagi, Japan. Tohoku J Exp Med.

[20] Silva RN, Duarte DA, de Oliveira AM (2020). The influence of television on the food habits of schoolchildren and its association with dental caries. Clin Exp Dent Res.

[21] Ghimire N, Rao A (2013). Comparative evaluation of the influence of television advertisements on children and caries prevalence. Glob Health Action.

[22] Punitha VC, Amudhan A, Sivaprakasam P, Rathanaprabu V (2015). Role of dietary habits and diet in caries occurrence and severity among urban adolescent school children. J Pharm Bioallied Sci.

[23] Doichinova L, Bakardjiev P, Peneva M (2015). Assessment of food habits in children aged 6-12 years and the risk of caries. Biotechnol Biotechnol Equip.

[24] Zahara AM, Nur Ili MT, Nurul Y (2013). Dietary habits and dental caries occurrence among young children: does the relationship still exist?. Malaysian Journal of Medicine and Health Sciences.

